# Visuospatial perspective-taking in social-emotional development: enhancing young children’s mind and emotion understanding via block building training

**DOI:** 10.1186/s40359-022-00976-5

**Published:** 2022-11-12

**Authors:** Melissa Pearl Caldwell, Him Cheung, Sum-Kwing Cheung, Jian-Bin Li, Tik-Sze Carrey Siu

**Affiliations:** 1 Department of Early Childhood Education, The Education University of Hong Kong, 10 Lo Ping Road, Tai Po, New Territories Hong Kong; 2 Department of Psychology, The Education University of Hong Kong, 10 Lo Ping Road, Tai Po, New Territories Hong Kong

**Keywords:** Theory of mind, Emotion understanding, Visuospatial perspective-taking, Social-emotional development, Psychological perspective-taking, Block building

## Abstract

**Background:**

Theory of Mind (ToM) refers to the ability to represent one's own and others' mental states, and emotion understanding involves appropriately comprehending and responding to others' emotional cues in social interactions. Individual differences in mind and emotion understanding have been associated strongly with verbal ability and interaction and, as such, existing training for children's ToM and emotion understanding is mostly language-based. Building on the literature on embodied cognition, this study proposes that mind and emotion understanding could be facilitated by one's visuospatial experience in simulating other's frames of reference.

**Methods:**

This protocol consists of two training studies. Study 1 will examine if visuospatial perspective-taking training promotes ToM and emotion understanding. Participants will consist of 96 4.5-year-olds and will be randomly assigned to one of two training groups: the altercentric block building group (trained to be visuospatial perspective-takers), or the egocentric block building group (no visuospatial perspective-taking is involved). Study 2 will compare the engagement of visuospatial perspective-taking and verbal interaction in the development of mind and emotion understanding. Participants will consist of 120 4.5-year-olds. They will be randomly assigned to one of three training groups: the socialized altercentric block building (both visuospatial perspective-taking and verbal interaction), the parallel altercentric block building (visuospatial perspective-taking only), or the paired dialogic reading (verbal interaction only).

**Conclusions:**

In terms of theoretical implications, the potential causal relationship between visuospatial perspective-taking and ToM and emotion understanding may shed new insights on what underlies the development of mental state understanding. The findings of this study also have practical implications: researchers and educators may popularize visuospatial perspective-taking training in the form of block-building games if it is found to be effective in complementing conventional language-based theory-of-mind training.

## Background

Theory of Mind (ToM) is the ability to ascribe mental states, such as desires, intentions, beliefs, or feelings, to oneself and others in social interactions [1]. Children recognize that the mental states of others may differ from their own when they acquire ToM. As such, children with ToM interact with others based on their interpretation of mental states of the other people. ToM and emotion understanding are related to one another and are conceptually interrelated [2, 3]. Emotion understanding involves comprehending and responding to emotional cues appropriately in social interactions [4]. Children's early social relationships are essential to developing ToM and emotion understanding [5], and children exposed to a conversation-rich environment have improved ToM and emotion understanding [6–8]. Ontogenetic studies show that the acquisition of ToM emerges between the ages of 3 and 5 [9, 10], but individual differences in ToM development often occur, and these individual differences in ToM are strongly associated with verbal ability and interaction [5, 11, 12].

Building on the literature on embodied cognition and visuospatial perspective-taking, this study proposes that mind and emotion understanding could be facilitated by one's visuospatial experience in simulating the frames of reference of others. This proposal consists of two visuospatial perspective-taking training studies:

1. Study 1 aims to demonstrate that visuospatial perspective-taking training promotes ToM and emotion understanding development in preschoolers.
2. Study 2 aims to reproduce the results of Study 1, and more importantly, to elucidate and compare the engagement of visuospatial perspective-taking and verbal interaction in the development of mental state understanding.

### Theory of mind and emotion understanding

Early studies on ToM development have been based on an either-or assumption. Such studies use false belief tasks to illustrate that a fully developed ToM is attained at around four years of age [9, 10]. More recently, researchers have considered the development of ToM to be a spectrum of mind understandings that occur over the course of development; ToM abilities unfold by successive insights that range from understanding of simple desires to more complex mental stances and perspectives [13–16]. On the basis of the spectrum of mental state understandings, individual differences in ToM among children become apparent [12, 17]. Even adults can make egocentric errors; past research has shown that adults may fail to resist interference from their own perspective when understanding the mental states and interpreting the behaviors of others [18, 19]. In the context of a communication game, bilingual adults’ ability to assume others’ mental states is also modulated by the cultural frame currently active in their mind [20]. These findings have sparked research into what makes individuals different when it comes to understanding the minds and emotions of others.

Emotion understanding involves appropriately comprehending and responding to emotional cues in social interactions [4]. Existing evidence shows that ToM and emotion understanding are related to one another, and are conceptually interrelated [2, 3, 21–23]. Longitudinal studies have revealed the relationship between ToM and emotion understanding, as well as the direction of influence between these two domains of social cognition: O’Brien and colleagues [24] investigated the relationship between ToM and emotion understanding and found that early emotion understanding at 3 years of age predicted later ToM performance at 4 years of age. Similarly, a recent longitudinal study by Kuhnert et al. [3] found significant associations between emotion understanding at 5 years and ToM at 7 years. These studies suggest that children’s emotion understanding precedes and supports their development of ToM.

Although emotion understanding occurs before ToM, ToM also contributes to children’s emotion understanding in social occasions. According to Eggum et al. [25], ToM appears to facilitate the understanding of the emotions of others, especially when the child’s emotional experiences differ from those of others. For example, ToM may assist a child who enjoys swimming in understanding that other children may be afraid of the water and experience fear in a pool. Children often draw on their ToM insights to assess other’s emotional responses.

### Theory of mind and language

Language ability has been consistently found to be associated with ToM performance [11, 26–30]. In particular, children’s understanding of mental state vocabulary, such as *think, believe, want, know*, and sentential complements such as “John believes/says it is raining” are linked to their ToM scores [29, 31–33]. There have been two main explanations for this relationship between ToM and language ability [11]. From the perspective of linguistic determinism, researchers argue that language plays a causal role in the development of ToM ability. In other words, children who lack language ability do not achieve ToM [31, 34]. However, there is much controversy over language's specific role in ToM development [26]. The domain-general perspective argues that the majority of the ToM tasks are verbal tasks and thus language is heavily needed in mental state understanding [35, 36].

A number of social-environmental factors have been found to be associated with children’s ToM development, including family size and the number of siblings [37–39], socioeconomic status [2], child’s sociolinguistic awareness [40], mother’s mind-mindedness [41], and mother–child mental state talk [6, 8]. These factors create a social-linguistically enriched environment for children to explore others’ mental stances and perspectives, and as such, children can respond appropriately to social interactions by inhibiting their egocentric perspective. Linguistic scaffolding from elder siblings and parents thus strengthens a child’s capacity to apprehend the abstract mind. From this, we can see that language ability and verbal interaction are essential to developing mind understanding, such as the ability to relate the actions of others to their mental states. This may contribute to the fact that existing ToM training is heavily language-based [42–44]. These language-based ToM training were explicitly designed to teach children mentalistic language and knowledge of inner states through role-playing and story-telling.

Hofmann et al. [43] conducted a meta-analysis of 45 language-based training studies that aim to improve children’s ToM performance. The meta-analysis showed that the aggregate effect size based on the comparisons of the 45 studies was moderately strong compared with the control group. Numerous studies in the meta-analysis appeared to have a significant impact on children’s ToM development, including having an explicit conversation about the mental states of others and emotion understanding [45, 46]. The research showed that explicit instruction could be used to teach and enhance one’s theory of mind [43]. Hofmann and colleagues emphasized the role of language in children’s ToM development, specifically, the importance of language in drawing children’s attention to mental states understanding. Consistent evidence has suggested that children’s language ability is associated with their ToM ability [5, 12, 26, 47]. However, the mechanisms underlying this relationship are still under debate. In the light of this, this paper proposes a novel view using the perspective of embodied cognition to address the competing theoretical accounts of ToM development.

### Embodied cognition

Embodied cognition is the idea that abstract mental representations are grounded in concrete sensorimotor experiences drawn from our bodily states, motions, and interactions with the surrounding environment [48–50]. Or, to put it another way, individuals perceive the world through their sensorimotor experience [51]. Previous studies have shown that individuals' current bodily experiences can influence higher cognition, such as thinking, reasoning, and evaluation during decision-making [52–54]. Empirical evidence supports this notion of the workings of the mind: In Williams and Bargh’s [55] research, participants experienced increased physical warmth or cold by holding a warm or cold object. It was the found that participants who had felt physical warmth were more likely to judge a target individual to have a warm personality and choose a gift for a friend rather than for themselves, than participants who had held the cold objects. Another study by Chu and Kita [56] illustrated that gesturing assists spatial problem-solving. In a series of experiments, Topolinski and colleagues have demonstrated that sensorimotor experiences may contribute to one's complex cognitive process, such as memory, preference, and attitude e.g. [57–59]. These findings suggest that humans often use their bodies as a reference frame to process the perceived environmental stimuli.

The literature on embodiment offers new insights into what may promote ToM development. Embodied cognition assumes that individuals tend to think through their actual or representational sensorimotor experiences. As such, individuals' representations of the minds of others may be facilitated by adopting others' frames of reference, and imagining how the world looks to them. This cognitive process of representing others' visual experiences has been understood as visuospatial perspective-taking [60, 61]. In a recent study, Erle and Topolinski [62] investigated the connection between embodied cognition (i.e., thinking through sensorimotor experience) and embodied simulation (i.e., project mental processes based on sensation). The study found that mental alignment that induced visuospatial perspective-taking necessitated some embodied transformation. This connection occurred more often when there was an orientation disparity between the individual and the target agent.

### The role of visuospatial perspective-taking in mental state understanding

The acquisition of perspective-taking and the ability to understand others’ mental states constitutes a milestone in social-cognitive development in children [43, 63–65]. Researchers have distinguished two levels of perspective-taking [60, 61, 66]: Level-1 perspective-taking involves determining the visibility of an object for another person from different vantage points; Level-2 perspective-taking entails representing how the object looks from another person's viewpoint.

Visuospatial perspective-taking is similar to level-2 perspective-taking as it involves visualizing how the world appears to another person [62]. Individuals engage in this when their frame of reference differs from that of another [67, 68]. Research reveals that visuospatial perspective-taking becomes more difficult as the angular disparity between the viewpoint of an individual and that of others increases. In a visuospatial perspective-taking paradigm, participants were shown an avatar sitting at a table with two objects. They were instructed to determine whether the avatar should use the right or left hand to pick up one of the two target objects. The experiments revealed that the reaction time of visuospatial perspective-taking increases with larger angular disparity between the participant and the avatar [60, 69].

Building on the notion of embodied cognition, this study proposes that the representation of the mind and emotion of others, i.e., psychological perspective-taking, is grounded in the representation of others’ visuospatial frame, i.e. it is grounded in visuospatial perspective-taking. Indeed, a growing body of evidence supports the close linkage between visuospatial and psychological perspective-taking [70]. For instance, children with autism spectrum disorder exhibit deficits in both visuospatial perspective-taking and empathy (which involves psychological perspective-taking) [71, 72]. Previous research has also found that visuospatial perspective-taking correlates with the personal disposition of empathy and social skills [73–77]. Schurz et al. [78] conducted a meta-analysis of fMRI studies, revealing that overlapping brain regions were involved in visuospatial perspective-taking and theory of mind. In a series of experiments, Erle and Topolinski [62] illustrated that adult participants were more likely to adopt the random thoughts of the target after they were prompted to take the target's visuospatial perspective. Moreover, the results showed that imagining the target's visual frame also increased the perceived similarity and liking for the target. In a recent study, Tian et al. [79] demonstrated a relation between spatial ability and theory of mind in 3.5- to 4-year-olds, and importantly, such relation is mediated by the young children’s visuospatial perspective-taking ability. The authors argued that the psychological mechanism of taking others’ visuospatial perspective may be involved in theory-of-mind development.

If we take the above studies together, we can see that there is strong empirical evidence to argue that simulating the visual world of another person may foster the representation of other's mind and emotional states. As such, scholars have begun to debate the sequence of the development of visuospatial and psychological perspective-taking. And whilst Moll and Kadipasaoglu [80] have argued that understanding the simple mental states of others emerges earlier than visuospatial perspective-taking, there is generally more evidence that suggests otherwise. More scholars regard visuospatial perspective-taking as an essential stepping-stone for the fully developed theory of mind [71, 81, 82].

### The present study

This proposal draws on the literature of embodied cognition and visuospatial perspective-taking to explore alternative approaches to promote early mind and emotion understanding. The potential role of visuospatial perspective-taking in developing ToM and emotion understanding prompted us to design a visuospatial-based ToM training. The study aims to determine whether training young children’s visuospatial perspective-taking promotes their theory of mind and emotion understanding.

This study involves two training studies to investigate the causal relationship between visuospatial perspective-taking and mental state understanding. In study 1, children aged 4.5 years will be randomly assigned to participate in either (a) altercentric block building (experimental group) or (b) egocentric block building (control group). Experimenters will train children’s visuospatial perspective-taking through a series of block-building activities. Several studies have shown that block building fosters young children’s spatial development [83–85]. The training structure is to reproduce block figures because this task provides abundant age-appropriate experiences for children to grasp the visuospatial relations between objects. It should be noted that this study will specifically train young children to become visuospatial perspective-takers by requiring them to adopt the visual frame of their play partner (i.e., *altercentric block building*). In contrast, children in the control group will be required to reproduce the model figure that looks the same from their own viewpoint (i.e., *egocentric block building*). The partner’s perspectives are irrelevant; subsequently, children’s visuospatial perspective-taking will not be trained. Two research questions will be addressed in study 1:

1. Is children’s visuospatial perspective-taking associated with their ToM and emotion understanding?
2. Do children trained in the visuospatial perspective-taking group improve significantly more than the control children in ToM and emotion understanding after 6 weeks of training?

Previous studies have found that participating in block-building tasks enhances children’s general spatial and mathematics abilities [83, 84, 86, 87]. These two skills will be assessed as a manipulation check to ensure the training improves children’s relevant skills. In this way, any absence of effects will unlikely be attributable to manipulation failure.

The main goal of study 2 is to determine whether visuospatial perspective-taking or conventional verbal training is more effective in enhancing children’s mind and emotion understandings. ToM training has mostly been heavily language-based because the literature has identified language as a strong correlate of ToM development [42, 44]. Visible social interaction without language input does not promote false-belief understanding. Hence verbal interaction is regarded to be crucial to mind understanding [88]. Moreover, a study by Lohmann and Tomasello [44] involving 3-year-old children also found that training on the theory of mind with minimal linguistic interaction was ineffective in improving children’s false belief understanding. On the basis of the embodiment framework, study 2 will investigate whether simulating the visuospatial experiences of others alone, i.e. *without* the presence of verbal communication, is sufficient to enhance mind and emotion understanding. By doing this, study 2 will compare the training effect caused by visuospatial perspective-taking to the one caused by mere verbal interaction. In study 2, children aged 4.5 years will be randomly assigned to participate in either (a) *socialised altercentric block building* (both visuospatial perspective-taking and verbal interaction), (b) *parallel altercentric block building* (visuospatial perspective-taking only), or (c) *paired dialogic reading* (verbal interaction only). Dialogic reading will be served as a promising interactive approach because this training involves back-and-forth verbal exchanges between children. In addition, dialogic reading is easy to administer with preschool children. The differences between groups in ToM and emotion understanding post-training would indicate whether visuospatial perspective-taking or verbal interaction group will be more effective in fostering mental state understandings. Two research questions will be addressed in study 2:

(1) What is the difference between the effect of visuospatial perspective-taking vs. general verbal interaction training on improving preschoolers’ ToM and emotion understanding?

(2) Do children trained in the socialised altercentric block building group improve significantly more than children in the parallel altercentric block building and paired dialogic reading groups in ToM and emotion understanding after 6 weeks of training?

## Research plan and methodology

### Study 1: examining the role of visuospatial perspective-taking in developing theory of mind and emotion understanding

#### Participants

Participants will consist of 96 typically developing Cantonese speaking 4.5-year-olds (48 males; 48 females). The targeted age group is 4.5-year-old children because the ontogenetic development of both ToM and emotion understanding abilities typically emerges at around 4 to 5 years of age [89].

This study will investigate the effect of visuospatial perspective-taking training on children’s ToM and emotion understanding abilities by capitalizing on their mind-understanding differences at this age. Several meta-analyses have reported moderate to large effect sizes for ToM, emotion understanding, and relevant spatial training [43, 90, 91]. The effect size of this study can thus be reasonably estimated as medium-to-large. We used G*Power 3.1 to conduct an a priori power analysis to determine the required sample size. Results indicated that a total sample of 79 participants was required to achieve a power of 0.80 for detecting a medium-to-large effect (*f* = 0.32) at an alpha of 0.05 for a MANOVA (MANOVA: Repeated measures, within-between interaction). To account for a potential attrition rate of 20%, we propose to recruit 48 children per group, thus 96 children in total, via electronic fliers on local internet groups.

#### Design and procedures

This research proposal has gained approval from the Human Research Ethics Committee of [blinded university]. Written informed consent will be obtained from all participants' parents or legal guardians before the initiation of the study. Parents or legal guardians will be asked to complete a set of questionnaires on family demographics, children's characteristics and executive functioning. Prior to the training, children will first complete a pre-test in a quiet room at the University. Then, children will be randomly assigned to either the (a) egocentric block building or (b) altercentric block building training group. The training period will be six weeks, with a 1.5-h training session per week. The training will be conducted in a child-friendly lab at the University. After the training sessions are completed, the children will be tested with the same battery of measures in the post-test. Upon completion of the training, the participants' parents or legal guardians will receive a report of their child's performance and a gift voucher valued at HKD$200.

#### Measures

Raven’s Coloured Progressive Matrices (RCPM; [92]) is a non-verbal reasoning test that measures the general cognitive ability of young children aged 4 to 11 years. The RCPM consists of three sub-tests (Set A, AB, B); each subtest has 12 items. For each item, the administrator presents an incomplete design and children are asked to find the missing part of the design from six options to complete the design. The items are arranged in ascending order of difficulty in each subtest. The ascending difficulty levels also apply to the subtests, where Set A is the easiest and Set B the most difficult. A score of one will be given for each correct answer. The maximum possible score is 36, where higher scores indicate a higher level of nonverbal reasoning abilities.

The Bear/Dragon task [93] assesses the executive function of behavioural inhibitory control in young children. This task is a simplified version of the Simon Says task, in which children are required to selectively obey or ignore specific commands according to the rules given by the experimenter. Two stuffed animals, a bear and a dragon, will first be introduced to the children. Then the administrator will instruct the children to follow action commands such as “touch your head” given by the bear, and ignore the ones given by the dragon. After practice, there are 10 randomized trials of the bear and dragon commands. Children’s responses will be assigned a score from 0 to 3 in each dragon trial (0 = full movement, 1 = partial movement, 2 = wrong movement, and 3 = no movement). The maximum possible score is 15, where a higher score indicates better ability in behavioural inhibitory control.

The Hong Kong Test of Preschool Oral Language (Cantonese) (TOPOL-EV; [94]) assesses young children’s oral language abilities in the preschool period (i.e., from 2 years 6 months to 5 years 11 months of age). The expressive vocabulary subtest will be used in this study. The expressive vocabulary subtest consists of 58 items. In each item, the administrator presents one picture and children are required to name the displayed picture. Five categories of vocabulary are tested in this subtest, such as stating the objects or the actions. The items are arranged in ascending order of difficulty. A score of one will be given for each correct answer. The maximum possible score is 58 and higher scores indicate better knowledge of Cantonese expressive vocabulary.

The Test of Early Mathematics Ability-3 (TEMA-3) [95] measures early informal and formal mathematics skills in children aged from 3 to 8 years 11 months. The test consists of 72 age-based items, and the items for age 4 (i.e., 8 items) will be used in this study. Ryoo et al. [96] categorized the 72 items into different categories, and the items for age 4 were grouped into five categories, including:

1. Counting objects, e.g., 'How many stars did you count?'
2. Calculation, e.g., 'Make your tokens just like mine by adding up 1 + 1 tokens'
3. Set construction, e.g., 'Can you give me 5 tokens?'
4. Verbal counting, e.g., 'Counting tokens from 1 to 10'
5. Numeral literacy, e.g., 'What is this number?'

Each item will be scored with children’s binary response (i.e., fail = 0 or pass = 1). The maximum possible score is 8, where high scores indicate better early mathematics abilities.

The 3D Test of Spatial Assembly (TOSA) [87] measures the general spatial abilities of children aged 3 years and above. The TOSA consists of 7 test items. In each item, children are presented with a model assembly with different individual LEGO blocks. They are required to re-create the model with the same number of blocks given to them. Each model has a base piece, i.e., the largest piece or the piece that is connected to the greatest number of other pieces, and component pieces, i.e., other pieces. Children are scored based on the accuracy of three aspects, including the correct vertical location of the pieces, the correct rotation of the pieces, and the correct placement of the pieces over block pips. The spatial test uses a two-step scoring system, including: (1) base piece scoring (i.e., pieces with spatial relationships and dependent on the base piece) and (2) subset scoring (i.e., pieces with spatial relationships and independent of the base piece). A score of 1 is given for every correct assembly. The maximum possible score is 14 (7 items for each scoring step). A higher score indicates better spatial abilities.

The Perspective-Taking Test for Children (PTT-C) [97] assesses children's ability to represent the viewpoint of others. This test is based on the seminal Three Mountains Task by Piaget and Inhelder [98]. The PTT-C consists of 3 practice items and 18 testing items. Children are presented with a colour printout showing a toy photographer taking pictures of one or two 3D objects from different angles with each item. Then, children are asked to choose the picture among four options that the toy photographer would have taken. One of the pictures shows the correct view, and the other three are foils that include the same objects but with different orientations or spatial relationships. The items ascend in difficulty (one object to two objects) and vary in the angular disparities between the children and the toy photographer (0°, 90°, or 180°). A score of 1 is given to each correct response. The maximum possible score is 18. Higher scores indicate better visuospatial perspective-taking skills.

The Test of Emotion Comprehension (TEC) [99] assesses understanding of emotion in children aged 3 to 11 [100]. The TEC comprises nine subtests: recognition, external causes, desire, belief, reminder, hiding, mixed, and morality. This study will administer five of the subtests age-appropriate for 4.5-year-old children, including external cause (5 items), a score is given when at least 4 out of 5 are correct; desire (4 items), a score is given when all are correct; belief (1 item), reminder (1 item), and hiding (1 item), a score is given when it is correct. In each item, children are presented a cartoon scenario with a gender-matched protagonist. These story scenarios should elicit different emotional responses in the protagonist. Children are then given four cartoon faces, each representing an emotional state (happiness, sadness, anger, fear, or normal). They are asked to speculate the emotional state of the protagonist in response to the event. The maximum possible score is 5. Higher scores indicate better emotion understanding.

The Theory-of-Mind Scale (ToMS) [89] measures children’s ability to understand different mental states (e.g., desire, knowledge, belief). The ToMS consists of seven interactive tasks, including diverse desire, diverse belief, knowledge access, content false belief, explicit false belief, and hidden emotion. In each task, children are presented with a story with props (e.g., puppets, band-aid box, toy carrot) and are asked questions regarding their own mental states and those of the target protagonists. A score of one is given to each correct answer. The maximum possible score is 5 and a higher score indicates a more advanced level of mental state understanding.

#### Training activities

The children will be randomly grouped into pairs, and a trained experimenter will guide them. All the experimenters will receive a three-hour training on the block-building training protocol. The training sessions will be videotaped and carefully reviewed; the experimenters will be given corrective feedback to ensure consistency of the instructions and guidance given to different pairs of children. Training trials vary in model complexity (2, 3, or 4 blocks in the model figure) and in seating position (see Table 1).

**Table 1:**
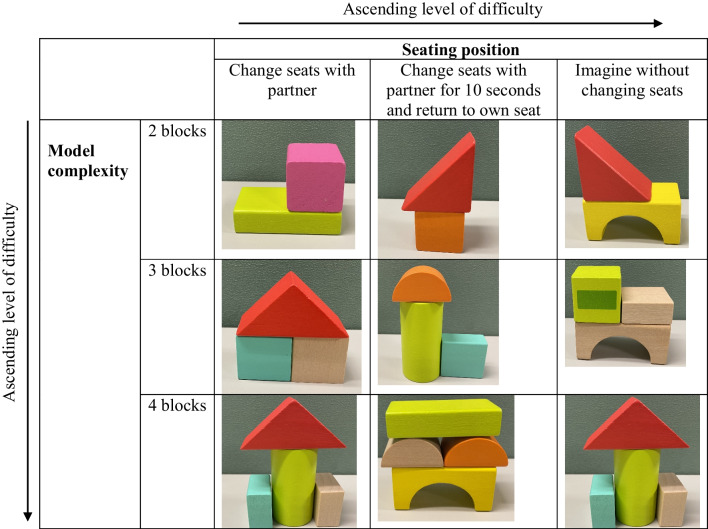
The gradation of difficulty in block-building training in study 1

Three types of seating positions exist during the *altercentric frame* time in the block-building training, including:

1. change seats with the partner and reproduce the model figure from the partner’s visual frame
2. change seats with the partner for ten seconds and return to the original seat to reproduce the model figure based on the partner’s visual frame
3. imagine how the model figure looks from the partner’s viewpoint and reproduce the model figure based on the partner’s visual frame.

The block-building training activities are described below:

**Table:** 

*Egocentric block building *training (control group; Fig. 1)	The block building training follows the training procedures from Verdine et al. [87]. The children are seated next to each other at the play table. The experimenter then presents a model figure built from blocks and places it at the center of the play table. The training begins with the *egocentric frame* time, in which children are instructed to reproduce the same block figure from a group of scattering blocks based on their own visual frame. The *altercentric frame* time follows, in which children are instructed to reproduce the block figure that looks the same from their partner’s point of view. Since the participant’s partner sits next to them, the block figure should look largely the same to the participant’s line of sight. The difficulty of building these block figures is age-appropriate for children aged 3–5 years [87]
(b) *altercentric block building* training (experimental group; Fig. 2)	In this training group, the children are seated opposite to each other at a play table. The training begins with the *egocentric frame* time, with the same procedure as the *egocentric frame* time of training (a). The *altercentric frame* time follows, during which the children try to adopt the visual frame of their play partner who is sitting on the opposite side of the play table. They will be instructed to imagine how the model figure looks for their partner and build a figure that looks the same from their partner's viewpoint. Since the participant’s partner seats opposite to them, the figure should have 180° angular difference to the participant’s line of sight

**Fig. 1:**
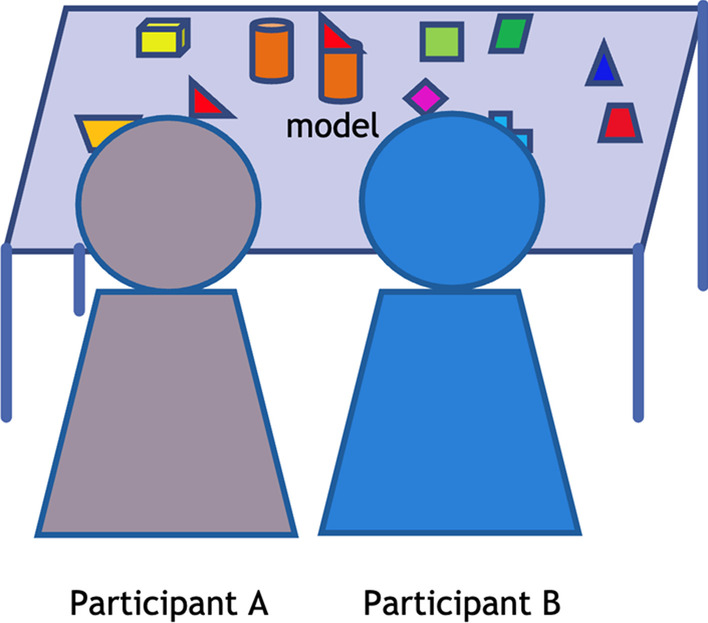
Control group: Egocentric block building training

**Fig. 2:**
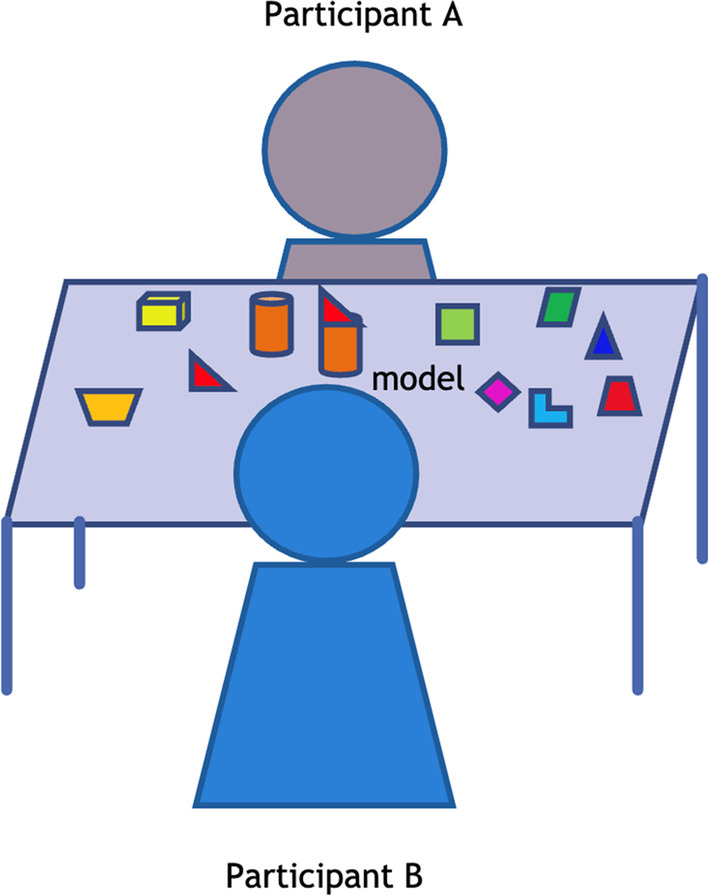
Experimental group: Altercentric block building training

#### Data analysis plan

First, we will perform correlation analyses with the pre-test data to examine the associations among visuospatial perspective-taking and ToM and emotion understanding. We hypothesise that there will be positive relationships among preschoolers' visuospatial perspective-taking, ToM, and emotion understanding.

We then will use independent *t*-tests to test the group differences in nonverbal reasoning, inhibitory control, and oral language proficiency and decide if these three variables will be used as covariates in subsequent analysis. Next, we will use repeated measures ANOVAs to examine the post-training improvements in general spatial and mathematics skills in both groups. Previous research has illustrated a positive effect of block-building on preschoolers’ spatial and mathematics skills [83, 84, 86, 87]. We will also examine if the altercentric block-building group improves significantly more than the egocentric block-building group in visuospatial perspective-taking. This will ensure that the altercentric training increases visuospatial perspective-taking that we aim to train.

In the main analysis, we will use repeated measures MANOVAs to determine the post-training changes in understanding mind and emotion across the two groups. We predict that the altercentric block-building group will show greater improvements in ToM and emotion understanding abilities than the egocentric block-building group. Moreover, these improvements will be mediated by the improved visuospatial perspective-taking ability.

### Study 2: Comparing the roles of visuospatial perspective-taking and verbal interaction in developing theory of mind and emotion understanding

#### Participants

This study will compare the effects of three different training methods on the development of mind and emotion understanding in preschool children. Participants will consist of 120 typically developing Cantonese-speaking 4.5-year-olds (60 males; 60 females), with 40 children in each training group. We used G*Power 3.1 to conduct an a priori power analysis to determine the required sample size. Results indicated that a total sample of 98 participants was required to achieve a power of 0.80 for detecting a medium-to-large effect (*f* = 0.32) at an alpha of 0.05 for a MANOVA (MANOVA: Repeated measures, within-between interaction). To account for a potential attrition rate of 20%, we propose to recruit 40 children per group, thus 120 children in total, via electronic fliers on local internet groups.

#### Design, measures and procedures

The procedures will be similar to those in study 1. Written informed consent and information on family demographics, child characteristics, and executive functioning will be obtained from all participants’ parents or legal guardians prior to the initiation of the study. Children will be pre-tested and post-tested with the same battery of measures as those used in study 1. The training period will also be six weeks, with one 1.5-h training session per week. The training will be conducted in a child-friendly lab at the University. Upon completion of the training, the participants' parents or legal guardians will receive a report of their child's performances and a gift voucher valued at HKD$200.

#### Training activities

The children will be randomly grouped into pairs, and a trained experimenter will guide them. Like study 1, the experimenters will receive a three-hour training on the protocol, and their instructions and guidance will be regularly monitored. Children will be randomly assigned to one of three training groups, namely (a) *socialised altercentric block building*, (b) *parallel altercentric block building*, and (c) *paired dialogic reading*. Among the training groups, the socialised and parallel altercentric block building groups are based on the altercentric block building group in study 1. Both groups will also have the same gradation of difficulties in terms of model complexity and seating position as in study 1. The paired dialogic reading is derived from the conventional language-based training. The three different training activities are described below:

**Table:** 

Training groups	Training activities	Visuospatial perspective-taking	Verbal Interaction
(a) *Socialised altercentric block building* (Fig. 3)	Two children will be seated next to each other, and a toy bear will be seated at 180°, 90°, or 270° angular difference to the line of sight of the children. The experimenter will present a model figure built from blocks. Children will be asked first to reproduce the figure based on their own visual frame. Then, they will be asked to adopt the visual frame of the toy bear and reproduce the figure. Note that the children will be encouraged to discuss the perspective of the toy bear when building the block-figure. Therefore, both visuospatial perspective-taking and verbal interaction will be trained in this group	✓	✓
(b) *Parallel altercentric block building* (Fig. 3)	This training group will have the same procedures as that in the (a) socialised altercentric block building group, except children in this group are required to reproduce the block-figure without discussing the perspective of the figurine. Therefore, only visuospatial perspective-taking will be trained in this group	✓	✗
(c) *Paired dialogic reading*	The storytelling intervention by Ornaghi et al. [101] will be adapted in this training group. The experimenter will read the book series "The Adventures of Jack and Theo" [102] to the children. Children will listen to three stories in each session. After each story, children will be engaged in different language games and conversations. This training will minimize the use of the mental state lexicon to avoid perspective-taking and mentalising. No block-building activity will be involved in the training group. Therefore, only verbal interaction will be trained in this group	✗	✓

**Fig. 3:**
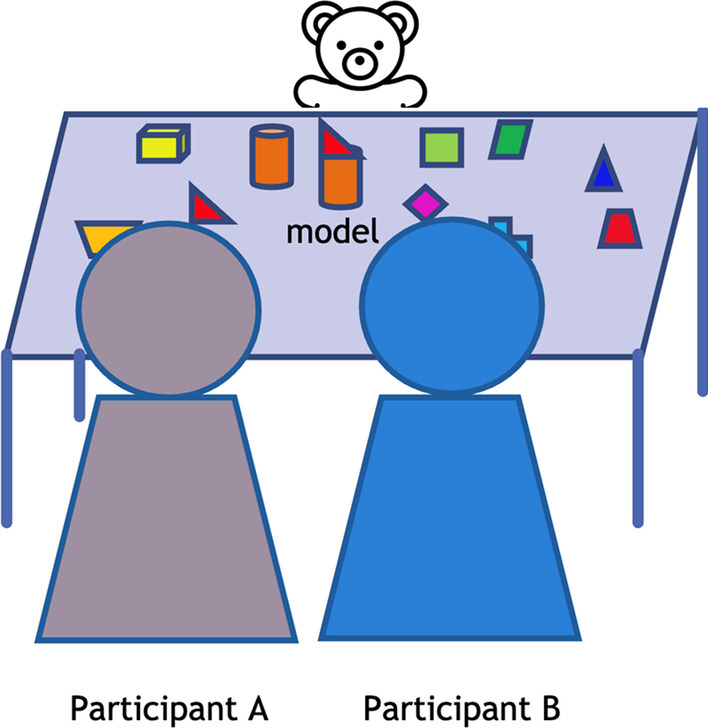
Socialised Altercentric Block Building Group and Parallel Altercentric Block Building Group

#### Data analysis plan

The preliminary analyses will be the same as those in study 1. Next, repeated measures MANOVAs will be conducted to compare the effectiveness of the three trainings in improving children’s visuospatial perspective-taking, ToM, and emotion understanding abilities. We predict all of the training in study 2 will increase children’s ToM and emotion understanding. In particular, we hypothesise that the *socialised altercentric block building* training will result in the greatest improvement on the children’s ToM and emotion understanding. We expect the paired dialogic reading to enhance children’s general language ability but not their visuospatial perspective-taking ability. In contrast, we expect that the *parallel altercentric block building* will enhance the children’s visuospatial perspective-taking ability but not their general language ability. We further hypothesise that the *socialised altercentric block-building* training will enhance both visuospatial perspective-taking and language abilities because verbal interaction may strengthen children’s developing ability to simulate the visuospatial experience of others. This finding will reveal whether visuospatial perspective-taking is conducive to developing ToM beyond the contribution of verbal interaction.

## Discussion

This study proposes a novel visuospatial-based block-building training to complement the conventional ToM training. The findings are expected to have theoretical and practical implications. Theoretically, this research may advance our knowledge of the nature of mental state understanding in two ways: First, if our findings show that ToM and emotion understanding can be improved by training then this will give credence to the idea that individual variations in mental state understanding are not entirely the result of maturational or developmental cognitive processes, but are partially driven by social-environmental input. More notably, the proposed visuospatial-based training will be directly compared to the traditional language-based training, which addresses the contrasting theoretical accounts of social-cognitive development. To date, the dominant view has been that language and verbal interaction is the basis of ToM development. This study draws on the embodied cognition literature to explore the possibility that mind and emotion understanding may be influenced by our simulation of another person’s visuospatial experiences. The findings of this research may shed new light on how mind understandings develop and may further bridge the conceptually related but empirically isolated literature of ToM and visuospatial perspective-taking.

The study also has direct practical implications. There has been a wealth of evidence that early mental state understanding strongly predicts positive child outcomes, including social competence and academic performance [103–108]. As a result, researchers may collaborate with practitioners to develop evidence-based training to increase mind and emotion understanding. To date, ToM training has predominantly relied on verbal interaction. ToM training generally involves specific training in language (such as understanding sentential complements and mental state terms) or explicit conversation regarding mental states and the relationship to behaviour. Paradoxically, ToM interventions often target children with autism spectrum disorder (ASD), who commonly have oral language deficits. This place children with ASD under challenging circumstances. In that light, the present study proposes new avenues to train psychological perspective-taking, supposing the results will indicate that visuospatial perspective-taking in the form of block building is a feasible and effective training paradigm. In that case, this specific block-building training can be introduced to complement the conventional ToM training. Block play is popular among young children, especially children with ASD. The proposed block-building activities ought to be attractive to them. Moreover, as blocks are always accessible in kindergarten classrooms, this proposed block-building training may be implemented in regular early childhood education settings to improve young children’s ToM and emotion understanding.

## Data Availability

Not applicable.

## Abbreviations

ANOVA: The one-way analysis of variance
ASD: Autism spectrum disorder
fMRI: Functional magnetic resonance imaging
PTT-C: The perspective-taking test for children
RCPM: Raven’s Coloured Progressive Matrices
TEC: The Test of Emotion Comprehension
TEMA-3: The test of Early Mathematics Ability-3
ToMS: The Theory-of-Mind Scale
ToM: Theory of Mind
TOPOL-EV: The Hong Kong Test of Preschool Oral Language (Cantonese)-Expressive vocabulary
TOSA: The 3D Test of Spatial Assembly
